# A multicenter prospective study of TACE combined with lenvatinib and camrelizumab for hepatocellular carcinoma with portal vein tumor thrombus

**DOI:** 10.1002/cam4.6302

**Published:** 2023-06-30

**Authors:** Xiaomi Li, Xiaoyan Ding, Mei Liu, Jingyan Wang, Wei Sun, Ying Teng, Yawen Xu, Hongxiao Wu, Wendong Li, Lin Zhou, Jinglong Chen

**Affiliations:** ^1^ Department of Cancer Center, Beijing Ditan Hospital Capital Medical University Beijing China; ^2^ Department of Oncology, Beijing You'an Hospital Capital Medical University Beijing China; ^3^ Department of Interventional Radiology, The Fifth Medical Center Chinese PLA General Hospital Beijing China

**Keywords:** camrelizumab, hepatocellular carcinoma, lenvatinib, portal vein tumor thrombus, transarterial chemoembolization

## Abstract

**Background and aims:**

Hepatocellular carcinoma (HCC) with portal vein tumor thrombus (PVTT) predicts a poor prognosis. The aim of the present study was to evaluate the efficacy and safety of using lenvatinib and camrelizumab combined with transarterial chemoembolization (TACE) to treat HCC with PVTT.

**Methods:**

This was a single‐arm, open‐label, multicenter, and prospective study. Eligible patients with advanced HCC accompanied by PVTT were enrolled to receive TACE combined with lenvatinib and camrelizumab. The primary endpoint was progression‐free survival (PFS), while the secondary endpoints included objective response rate (ORR), disease control rate (DCR), overall survival (OS), and safety.

**Results:**

Between April 2020 and April 2022, 69 patients were successfully enrolled. With a median follow‐up time of 17.3 months, the median age of the patient cohort was 57 years (range: 49–64 years). According to modified Response Evaluation Criteria in Solid Tumors, the ORR was 26.1% (18 partial responses [PRs]) and the DCR was 78.3% (18 PRs, 36 stable diseases [SDs]). The median PFS (mPFS) and median OS (mOS) were 9.3 and 18.2 months, respectively. And tumor number >3 was identified as an adverse risk factor for both PFS and OS. The most common adverse events across all grades included fatigue (50.7%), hypertension (46.4%), and diarrhea (43.5%). Twenty‐four patients (34.8%) experienced Grade 3 toxicity that was relieved by dose adjustment and symptomatic treatment. No treatment‐related deaths occurred.

**Conclusions:**

TACE combined with lenvatinib and camrelizumab is a well‐tolerated modality treatment with promising efficacy for advanced HCC with PVTT.

## 1 INTRODUCTION

Primary liver cancer (PLC) represents the sixth most common form of cancer and the third leading cause of cancer death worldwide, and hepatocellular carcinoma (HCC) accounts for 75–85% of cases of PLC.^1^, ^2^ Around 44%–62% of HCC patients present with portal vein tumor thrombus (PVTT) that plays a major role in disease prognosis.^3^, ^4^ Both National Comprehensive Cancer Network and European Society for Medical Oncology guidelines suggest that patients with HCC and PVTT should be classified as Barcelona Clinic Liver Cancer stage C (BCLC‐C) and receive systemic treatment.^5^, ^6^ Despite advancements in understanding the molecular etiology of HCC, the outcomes for HCCs with PVTT remain unsatisfactory, and the optimal treatment modality for such patients has not been established.

For unresectable HCC, transarterial chemoembolization (TACE) is one of the most commonly performed techniques and can significantly improve survival.^7^, ^8^, ^9^, ^10^ Because of the synergic effect, TACE combined with sorafenib could prolong the time to progression (TTP) in HCCs with PVTT of the first‐order or lower‐branch, albeit for no more than 2 months.^11^, ^12^, ^13^ The REFLECT trial showed that lenvatinib improved TTP and objective response rate (ORR) compared to sorafenib, but this study did not include HCC with PVTT type III/IV.^14^ Several studies have also shown better results using TACE and lenvatinib for HCC with PVTT compared to TACE and sorafenib.^15^, ^16^, ^17^

Recently, increasing evidence suggests that immune checkpoint inhibitors (ICIs) combined with either multi‐kinase tyrosine kinase inhibitors (TKIs) or vascular endothelial growth factor (VEGF) antibody become the trending regimens for advanced HCC.^18^, ^19^, ^20^, ^21^ Moreover, inflammatory factors generated and released during TACE treatment have a priming effect on adaptive immunity; TACE can induce spontaneous T‐cell responses and has regulatory effects on the tumor microenvironment. Ultimately, the combined use of TACE and ICIs may be more effective in promoting antitumor immune reconstitution.^22^ Two retrospective studies demonstrated that TACE combined with camrelizumab and TKIs controlled tumor progression and prolonged survival.^23^, ^24^ However, limited data of this triplet regimen on HCC with PVTT are available. The above studies thus prompted us to test the efficacy and safety of using TACE combined with lenvatinib and camrelizumab for treating HCC with PVTT.

## 2 METHODS

### 2.1 Patient cohorts and data availability

This study was designed as an open‐label, multicenter, prospective study. Patients with a clinical diagnosis of HCC based on histological examination or American Association for the Study of Liver Diseases (AASLD) guidelines, irrespective of PVTT types, were enrolled in this study. Other inclusion criteria were: (1) age 18–75 years; (2) at least one measurable lesion defined by modified Response Evaluation Criteriain Solid Tumors (mRECIST); (3) ECOG‐PS 0 or 1; (4) Child–Pugh score ≤7; (5) adequate cardiac, hepatic, renal, bone marrow, and hematologic function; and (6) predicted life expectancy >12 weeks. Key exclusion criteria included: (1) uncontrolled hypertension; (2) active autoimmune diseases; (3) combined with other untreated malignancies; (4) pregnancy or lactation; (5) HCC with complete portal vein trunk obstruction; or (6) prior history of systemic and locoregional therapy for PVTT. Although subjects were allowed to have extrahepatic tumor spread, brain metastases or complete obstructive invasion of the primary branches of the biliary duct need to be ruled out. PVTT type was defined by Cheng‘s classification, Type I refers to tumor thrombus distant to the second or second grade branch of the portal vein, Type II refers to tumor thrombus found in the left or the right branch of the hepatic portal vein, Type III refers to tumor thrombus in the main portal vein lumen, and tumor thrombus found in the superior mesenteric vein is Type IV.

Written approval was obtained from the Institutional Review Board of Beijing Ditan Hospital, Capital Medical University before starting the study (BJDT CMU IRB; ethics code: JDLKZ 2021‐003‐02). The study was conducted in accordance with good clinical practice, with the principles outlined in the Declaration of Helsinki and local laws. All participants provided written informed consent prior to enrollment.

### 2.2 Study design

The prospective study was performed in three hospitals in Beijing from April 2020 to April 2022: Beijing Ditan Hospital, Capital Medical University; Beijing Youan Hospital, Capital Medical University; and The Fifth Medical Center of Chinese PLA General Hospital. According to the literature, the PFS of BCLC‐C HCCs treated with TACE combined with lenvatinib and ICIs is about 8 months, and the PFS of HCCs treated with TACE and lenvatinib is about 5 months. The PASS15.0 software was used, with α‐value = 0.05, β‐value = 0.1, time = 12 months, follow‐up = 12 months, and the calculated sample size = 64 cases.

### 2.3 Treatment

TACE was performed by two professional radiologists, and all patients underwent lipiodol‐based TACE. The chemoembolization emulsion was prepared by mixing the chemotherapeutic drugs (multiple components of adriamycin hydrochloride [20–60 mg] or oxaliplatin [50 mg], epirubicin [10 mg], and mitomycin [10 mg]) with 6–10 mL of lipiodol and an appropriate amount of contrast medium through the catheter. Some patients with rich tumors or arteriovenous shunts had solid embolic agents (300–500 μm) such as gelatin sponge or polyvinyl alcohol particles until the artery supplying the tumor was occluded and the tumor staining disappeared. TACE was performed on demand, subsequent TACE would be repeated when viable lesions or incomplete lipiodol uptake of liver tumor were demonstrated on the test of computed tomography (CT) or magnetic resonance imaging (MRI). The interval of repeat TACE was 8–12 weeks. Lenvatinib was administered orally at 12 mg/day for patients weighing ≥60 kg and 8 mg/day for patients weighing <60 kg, starting 7 days before the first cycle of TACE after enrollment. The drug was stopped on the day of TACE. The first dose of camrelizumab 200 mg was given intravenously on day 5 ± 2 after the initial TACE procedure and then every 3 weeks (Q3W). Both of lenvatinib and camrelizumab were continued until disease progression or intolerable toxicity. Dose reductions or interruptions were permitted for drug‐related adverse events.

### 2.4 Study evaluation

Efficacy assessments were performed every 8 weeks for the first 6 months and then every 12 weeks until disease progression or treatment discontinuation. Survival follow‐up was performed every 4 weeks until death, inability to follow‐up, or study completion. Tumor response was assessed according to mRECIST and classified as complete response (CR), partial response (PR), stable disease (SD), and progressive disease (PD). The initial tumor assessment was at week 8 based on radiographic images of contrast‐enhanced CT or MR. The best tumor response was recorded as the final results of evaluation. Progression‐free survival (PFS) was defined as the primary endpoint. The secondary endpoints included ORR, disease control rate (DCR), overall survival (OS), and safety. PFS was defined as the time from the date of the first dose of lenvatinib until disease progression or death from any cause. ORR was calculated as the percentage of patients with CR and PR. DCR was estimated as the percentage of patients with CR, PR, and SD. OS referred to the time from the start of treatment until death from any cause or last follow‐up. Safety was assessed and graded using CTC‐AE (Version 5.0).

### 2.5 Statistical analysis

Statistical analysis was conducted using R version 4.0.5. Continuous variables were expressed as mean ± standard deviation or median (interquartile range) and analyzed using the *T*‐test or Mann–Whitney *U*‐test. Categorical variables were presented in frequencies (proportions) and compared using the chi‐square test or Fisher's exact test. PFS and OS were calculated by the Kaplan–Meier method and the differences between groups were assessed via the log‐rank test. A univariable and multivariable Cox proportional hazards model was used to predict prognostic factors for PFS and OS. A value of *p* < 0.05 was considered statistically significant.

## 3 RESULTS

### 3.1 Patient characteristics

**FIGURE 1:**
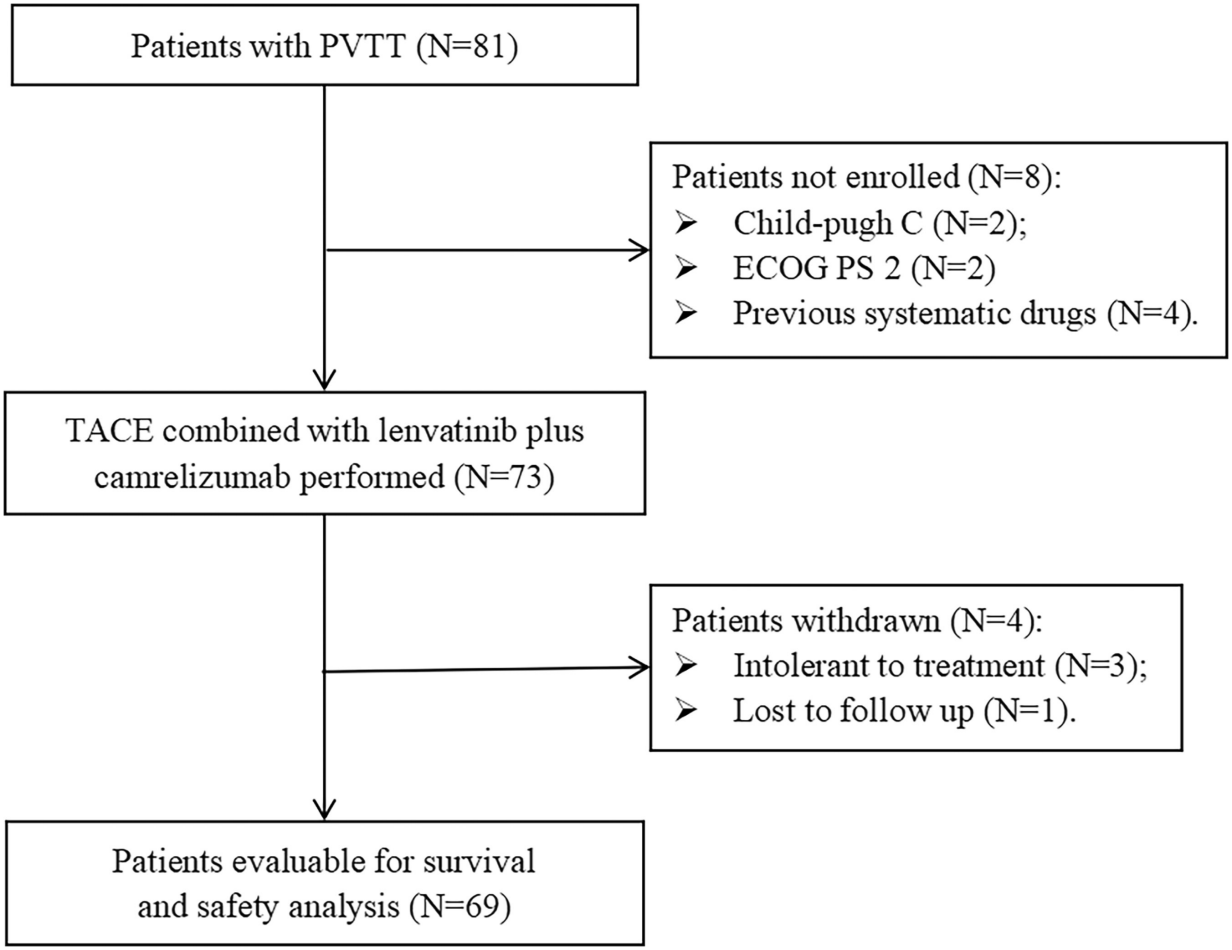
Flowchart of the present prospective trial. PVTT, portal vein tumor thrombosis; TACE, transarterial chemoembolization; ECOG PS, Eastern Cooperative Oncology Group performance status.

Between April 2020 and April 2022, a total of 81 HCC with PVTT were screened at three centers. Eight patients who had protocol violations (including two with Child–Pugh Grade C, two ECOG PS = 2, and four with previous systematic drugs) were excluded, and the remaining 73 participants received TACE combined with lenvatinib and camrelizumab. In addition, 3 patients were intolerant to treatment, 1 patient was lost to follow‐up, and finally 69 participants were enrolled, as shown in Figure 1. Beijing Ditan Hospital, Beijing Youan Hospital, and the Fifth Medical Center of Chinese PLA General Hospital included 34, 14, and 21 patients, respectively. The baseline characteristics are summarized in Table 1. The median age of the entire cohort was 57 years (ranging from 49 to 64 years). The majority of patients were male (78.3%; *n* = 54), predominantly infected with hepatitis B virus (HBV) (82.6%; *n* = 57); 72.5% of the patients had an ECOG PS score of 0 (*n* = 50). Eighteen (26.1%) patients had multiple tumors, and the median tumor diameter was 8 cm (ranging from 5.2 to 11.0 cm) in all cohorts. The number of patients with PVTT type I/II, and extrahepatic metastasis was 42 (60.9%), and 24 (34.8%), respectively. Alpha‐fetoprotein (AFP) was stratified at 400 ng/mL and 27 (39.1%) patients exceeded this cutoff. Most patients had good liver function, while 91.3% (*n* = 63) of patients had albumin–bilirubin (ALBI) class 1–2 and 60.9% (*n* = 42) had Child–Pugh Grade A.

**TABLE 1:** Patient demographics and baseline characteristics.

Characteristic	Group	Value
Median age, years [range]		57.0 [49.0, 64.0]
Sex, *n* (%)	Male	54 (78.3)
	Female	15 (21.7)
HCC etiology, *n* (%)	HBV	57 (82.6)
	HCV	3 (4.3)
	Alcohol	3 (4.3)
	Unknown	6 (8.6)
ECOG PS, *n* (%)	0	50 (72.5)
	1	19 (27.5)
Number, *n* (%)	≤3	51 (73.9)
	>3	18 (26.1)
Median size, cm [range]		7.9 [5.2, 11.0]
PVTT, *n* (%)	I	9 (13.0)
	II	33 (47.8)
	III	16 (23.2)
	IV	11 (15.9)
Metastastic sites, *n* (%)		24 (34.8)
	Lung	15 (21.7)
	Bone	5 (7.2)
	Peritoneal	2 (2.9)
AFP, *n* (%)	< 400 ng/mL	42 (60.9)
	≥400 ng/mL	27 (39.1)
Prior therapy, *n* (%)	Resection	7 (10.1)
	Ablation	26 (37.7)
ALBI, *n* (%)	1	11 (15.9)
	2	52 (75.4)
	3	6 (8.7)
Child‐Pugh grade, *n* (%)	A	42 (60.9)
	B	27 (39.1)

Abbreviations: AFP, alpha‐fetoprotein; ALBI, albumin‐bilirubin grade; ECOG PS, Eastern Cooperative Oncology Group performance status; HBV, hepatitis B virus; HCC, hepatocelluar carcinoma; HCV, hepatitis C virus; PVTT, portal vein tumor thrombosis.

### 3.2 Efficacy

**FIGURE 2:**
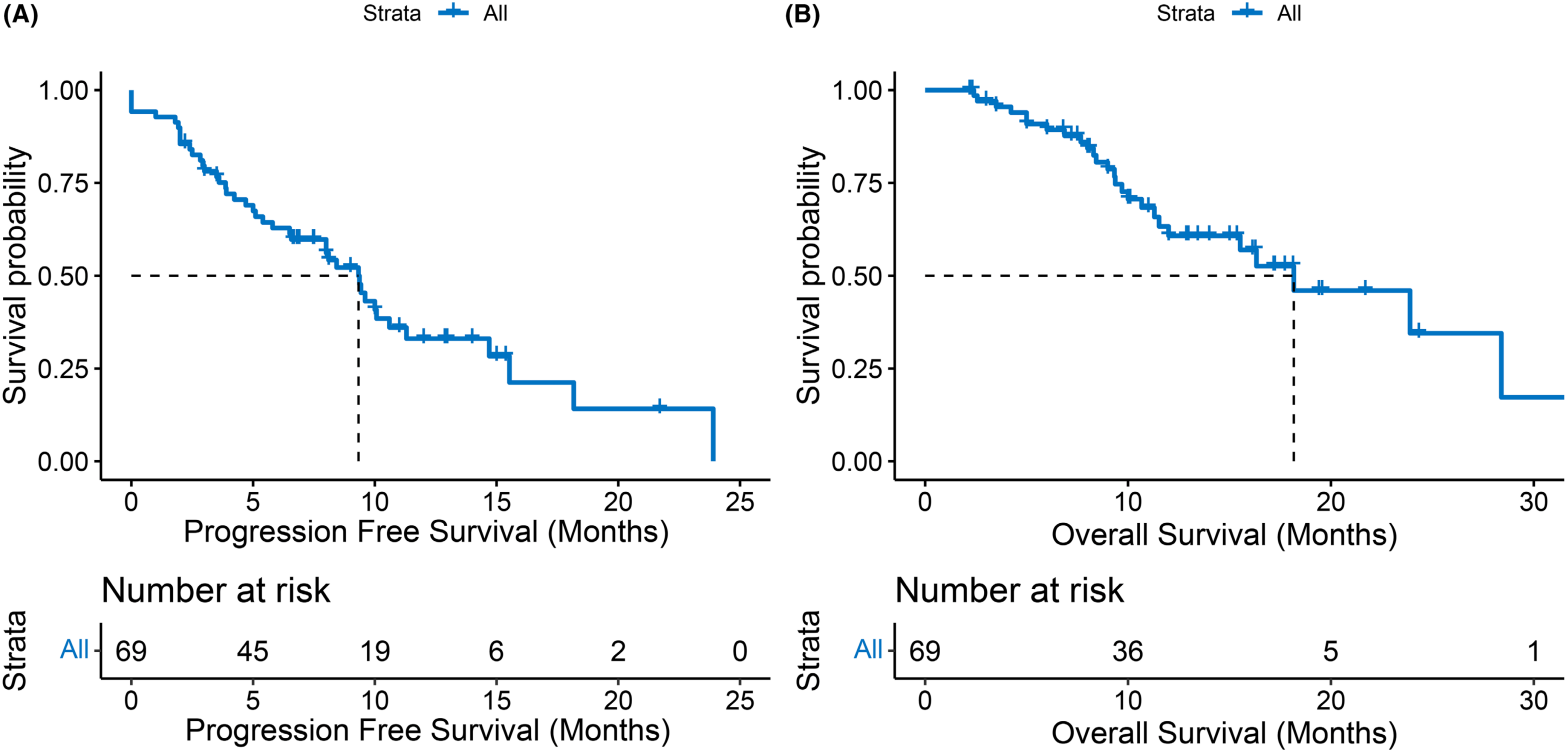
Kaplan–Meier curves of progression‐free survival (A) and overall survival (B) in the whole cohort.

**FIGURE 3:**
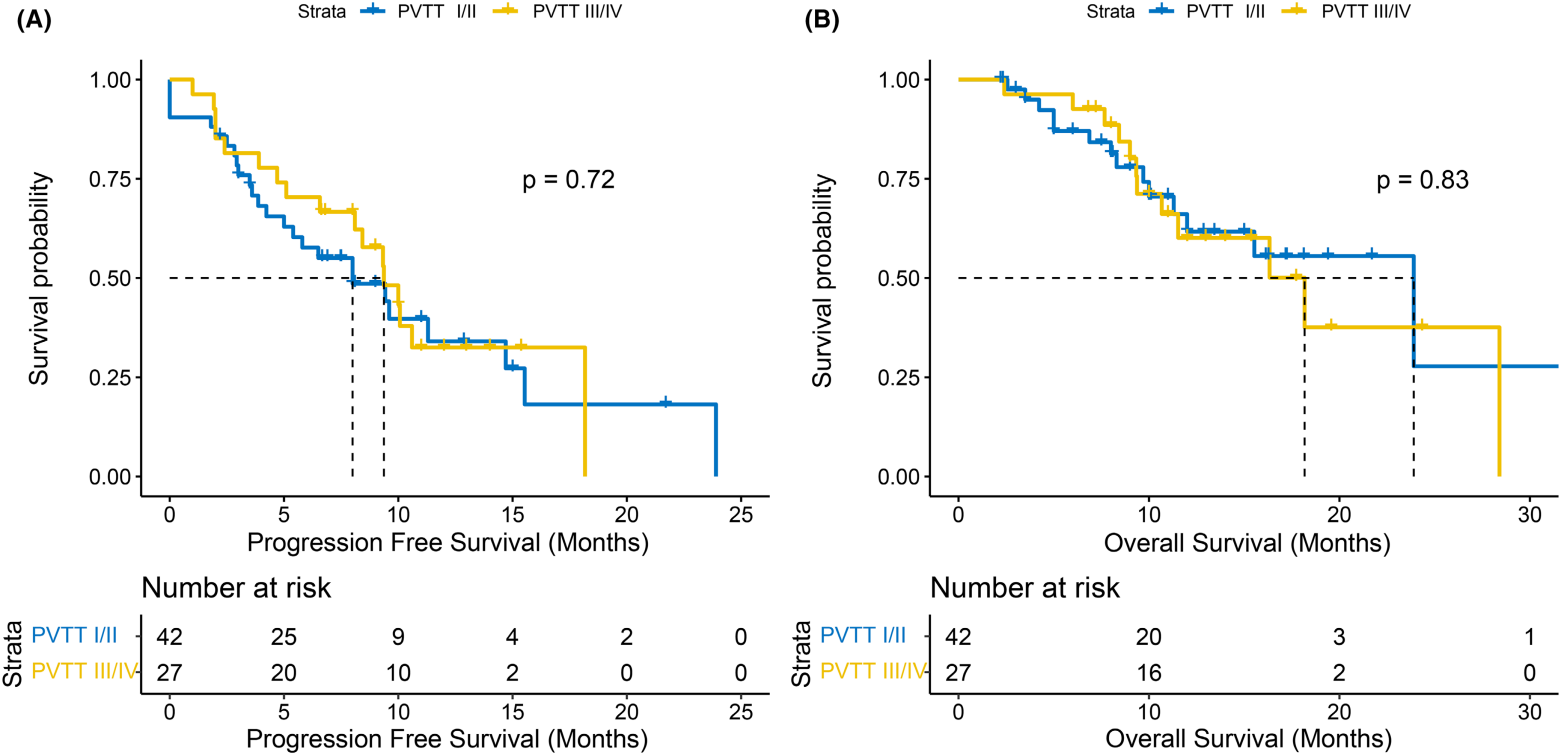
Kaplan–Meier curves of progression‐free survival (A) and overall survival (B) between PVTT type I/II and PVTT type III/IV. PVTT, portal vein tumor thrombosis.

The median follow‐up time was 17.3 months, during which 43 HCC patients progressed and 27 died. The median PFS was 9.3 months (95% confidence interval [CI], 6.6–11.3) and the median OS was 18.2 months (95% CI, 12‐not reach [NR]). The Kaplan–Meier curves of PFS and OS are shown in Figure 2A,B, respectively. When the prognosis of PVTT type I/II was compared with that of Type III/IV, neither PFS (8.0 months vs. 9.3 months, *p* = 0.72) nor OS (23.9 months vs. 18.2 months, *p* = 0.83) was significantly different (Figure 3A,B).

**FIGURE 4:**
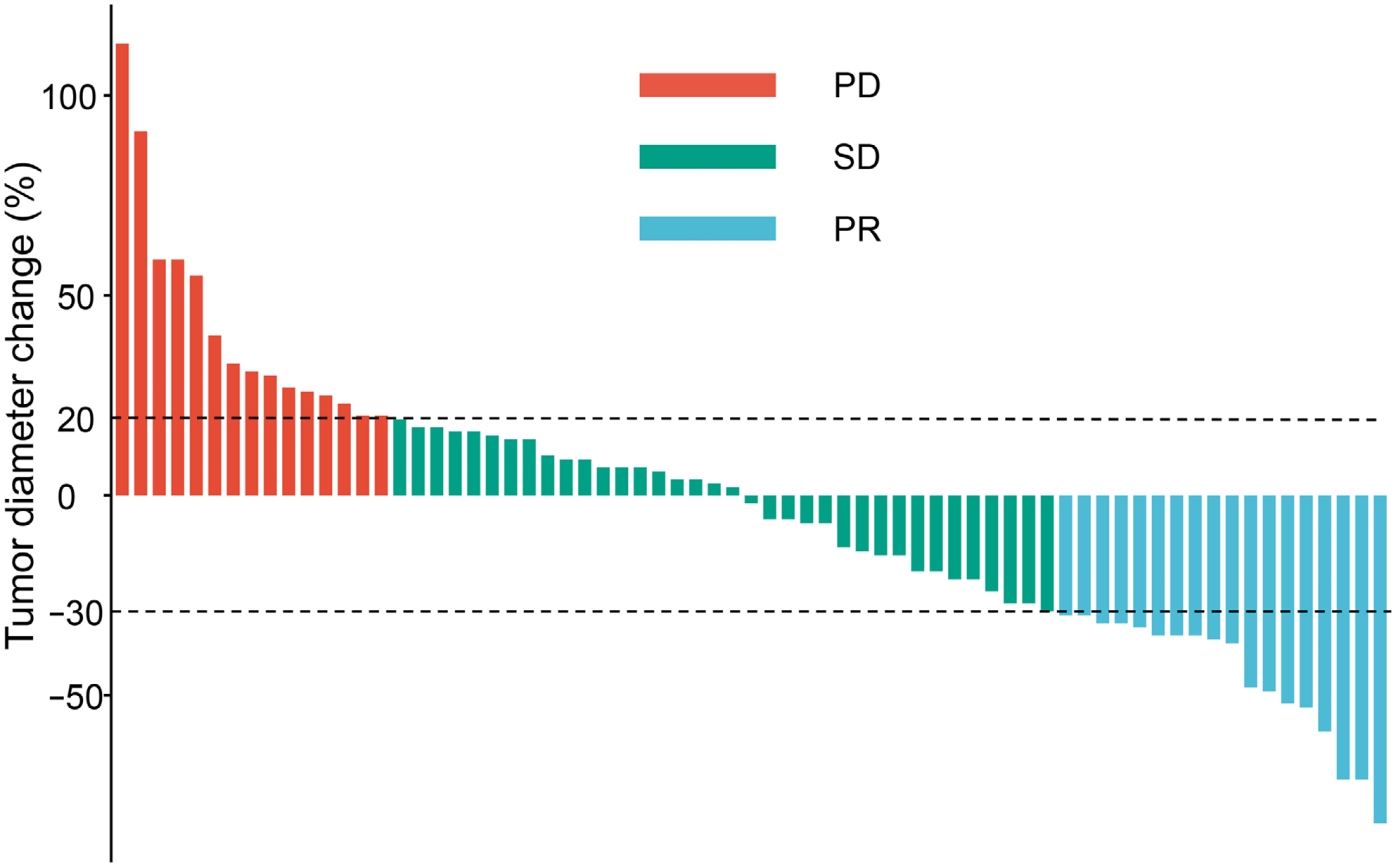
Waterfall plot of the changes in the target tumor diameter. PR: partial response; SD: stable disease; PD, progressive disease.

The waterfall plot of changes in tumor size was assessed according to mRECIST criteria (Figure 4): 18 (26.1%) PR, 36 (52.2%) SD, and 15 (21.7%) PD. The ORR of the comprehensive regimen was 26.1%, and the DCR was 78.3%. Of the 37 patients who progressed, 16 continued with lenvatinib and sintilimab, 9 did not change treatment, 5 received regorafenib, 2 used sorafenib, 1 was treated with donafinib, and the remaining 4 patients no longer used any other systemic drugs after progression.

**FIGURE 5:**
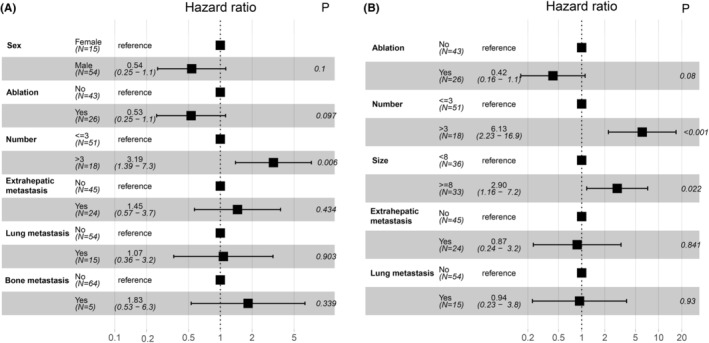
Forest plot for multivariate cox regression of progression‐free survival (A) and overall survival (B).

As depicted in Table 2, when we evaluated the effect of baseline characteristics on treatment response, we found that females, absence of ablation history, tumor number >3, and extrahepatic metastasis were associated with faster progression by univariate analysis; while absence of ablation history, tumor number >3, tumor size ≥8 cm and extrahepatic metastasis were associated with worse survival. Multivariate analysis was conducted for the above factors and showed that the number of tumors >3 (HR, 3.19; 95% CI, 1.39–7.3; *p* = 0.006) was identified as an independent adverse factor for PFS (Figure 5A). In addition, tumor number >3 (HR, 6.13; 95% CI, 2.23–16.9; *p* < 0.001) and tumor size ≥8 cm (HR, 2.90; 95% CI, 1.16–7.2; *p* = 0.022) were independent risk factors for death (Figure 5B).

**TABLE 2:** Univariate cox regression for PFS and OS.

Characteristic	PFS		OS	
	HR (95%CI)	*p*	HR (95%CI)	*p*
Sex (male vs. female)	0.48 (0.22–1.01)	0.054	0.7 (0.26–1.92)	0.489
Age (≥60 years vs. <60 years)	0.77 (0.4–1.46)	0.417	0.72 (0.32–1.65)	0.441
ECOG PS (0 vs. 1)	0.71 (0.34–1.46)	0.348	0.9 (0.37–2.18)	0.822
Resection (yes vs. no)	1 (0.42–2.41)	0.991	0.83 (0.25–2.78)	0.757
Ablation (yes vs. no)	0.42 (0.21–0.85)	0.016	0.37 (0.14–0.94)	0.036
Number (>3 vs. ≤3)	4.23 (2.11–8.47)	<0.001	4.91 (2.1–11.46)	<0.001
Size (≥8 cm vs. <8 cm)	1.53 (0.81–2.88)	0.191	2.85 (1.24–6.56)	0.014
PVTT (III/IV vs. I/II)	0.85 (0.46–1.57)	0.599	1.09 (0.5–2.36)	0.829
Extrahepatic metastasis (yes vs. no)	2.27 (1.21–4.27)	0.011	1.99 (0.89–4.44)	0.095
Lung metastasis (yes vs. no)	2.28 (1.16–4.46)	0.017	2.17 (0.93–5.08)	0.075
Bone metastasis (yes vs. no)	2.72 (1.05–7.02)	0.039	2.46 (0.73–8.31)	0.146
Child‐Pugh Grade (A vs. B)	1 (0.85–1.17)	0.991	1.07 (0.87–1.32)	0.525
AFP (≥400 ng/mL vs. <400 ng/mL)	1.28 (0.69–2.37)	0.441	1.85 (0.86–4.01)	0.118

Abbreviations: AFP, alpha‐fetoprotein; ECOG PS, Eastern Cooperative Oncology Group performance status; HR (95%CI), hazard ratio (95% confidence interval); OS, overall survival; PFS, progression‐free survival; PVTT, portal vein tumor thrombosis.

### 3.3 Safety

All adverse events related to treatment were demonstrated in Table 3. At least one AE occurred in 62 (89.9%) patients, and the most common AEs of any grade were fatigue (50.7%), hypertension (46.4%), diarrhea (43.5%), and decreased appetite (43.5%). The majority of the toxicities observed were mild to moderate. Grade 3 AEs were present in 24 (34.8%) patients, with 6 exhibiting hypertension, 3 obvious fatigue, 2 severe diarrhea, 2 decreased appetite, 2 elevated transaminase, 2 thrombocytopenia, 2 proteinuria, 2 hepatic encephalopathy, 1 hand and foot syndrome and 1 hyperbilirubinemia. In addition, there were no treatment‐related deaths during the study. Most treatment‐related AEs were relieved by dose reduction and symptomatic treatment. The immune‐related AEs (irAEs) were mainly reactive cutaneous capillary endothelial proliferation (RCCEP) in 10 cases, immune‐related hepatitis in 4 cases, interstitial pneumonia in 1 case and myocarditis in 1 case, all of which were mild to moderate except for two case of hepatitis that was Grade 3. The patient was diagnosed with Grade 3 immune‐related hepatitis, which was relieved without sequelae after a steroid administration of 4 weeks, and then he was re‐challenged with camrelizumab after 6 weeks. Immune‐related myocarditis was Grade 2, leading to permanent withdrawal of camrelizumab. Dose reductions occurred in 15 (21.7%) patients and drug interruptions in 5 (7.2%) patients; the reasons for dose reductions and discontinuation were shown in Table 3.

**TABLE 3:** Treatment related adverse events.

Adverse events	Any grade	Grades 3–4
All (Treatment‐related)	62 (89.9%)	24 (34.8%)
Fatigue	35 (50.7%)	3 (4.3%)
Hypertension	32 (46.4%)	6 (8.7%)
Diarrhea	30 (43.5%)	2 (2.9%)
Decreased appetite	30 (43.5%)	2 (2.9%)
Hand and foot syndrome	26 (37.7%)	1 (1.4%)
Hypothyroidism	14 (20.3%)	
Hyperbilirubinemia	10 (14.5%)	1 (1.4%)
Elevated transaminase	9 (13.0%)	3 (4.3%)
Nausea/vomiting	9 (13.0%)	
Thrombocytopenia	8 (11.6%)	2 (2.9%)
Proteinuria	8 (11.6%)	2 (2.9%)
Peripheral edema	3 (4.3%)	
Hepatic encephalopathy	2 (2.9%)	2 (2.9%)
Abdominal pain	1 (1.4%)	
Immune‐related AE (irAE)	16 (23.2%)	2 (2.9%)
Reactive cutaneous capillary endothelial proliferation	10	
Hepatitis	4 1	2
Interstitial pneumonia	1	
Myocarditis	1	
Dose reduction	15 (21.7%)	
Drug interruption	5 (7.2%)	
Hypertension	3	
Diarrhea	2	
Decreased appetite	2	
Fatigue	2	
Diarrhea	1	
Hepatic encephalopathy	2	
Grades 3 thrombocytopenia	2	
Interstitial pneumonia	1	

## 4 DISCUSSION

No optimal treatment modalities for HCCs with PVTT have been established yet, especially for those with Type III/IV PVTT.^25^, ^26^ And due to its poor prognosis and high risk of bleeding,^19^ there is no prospective study to investigate the triplet regimen of TACE with TKIs and ICIs. This study assessed the efficacy and safety of TACE combined with lenvatinib and camrelizumab in 69 HCC patients accompanied by PVTT. The results showed that the ORR was 26.1%, DCR 78.3%, mPFS 9.3 months, and mOS 18.2 months.

Non‐operative treatment is preferred for HCC with PVTT, especially the combination of two local treatments as well as local treatment combined with systemic treatment. Several studies have found that TACE combined with radiotherapy is more effective than TACE alone or sorafenib alone in such patients, but it has no superiority compared with the combination of TACE plus sorafenib.^27^, ^28^, ^29^, ^30^ Direct comparison between the triplet regimen of TACE combined with TKI plus ICI and the combination of TACE plus radiotherapy are lacking, and it needs to be further explored. Several studies have investigated the efficacy of TACE combined with TKI in HCC with PVTT, showing a median TTP/PFS of 3.1–8.4 months and OS of 7.5–16.4 months.^15^, ^16^, ^17^ Great progress has been made in current clinical trials on TKIs with ICIs for advanced HCC. The phase III IMbrave150 trial showed that bevacizumab with atezolizumab significantly prolonged the PFS (6.8 months) and OS (19.2 months).^19^ The phase Ib KEYNOTE‐524 trial found that the PFS was 8.6 months and the OS was 22 months in the lenvatinib and pembrolizumab double‐regimen group, and the phase III LEAP‐002 is ongoing.^18^ Although there are few studies on TACE with target‐immune combination in HCC patients with PVTT, the above data suggest the great clinical prospect of the triple regimen and further studies are warranted.

The robust antitumor efficacy has revealed a potential mechanism of the triplet regimen. TACE may cause hypoxia to activate hypoxia‐inducible factor‐1a (HIF‐1a), which in turn regulates other pro‐angiogenic factors, such as VEGF.^31^, ^32^, ^33^ Thus, TACE stimulates tumor angiogenesis and promotes survival, growth and metastasis of tumor cells.^31^ The addition of anti‐angiogenic drugs to the existing TACE regimen may counteract the induced angiogenesis and improve the prognosis of advanced HCC.^33^ In addition, TACE has a regulatory effect on the tumor microenvironment, and combined immunotherapy may enhance the efficacy.^22^ For instance, VEGF receptor inhibitors can enhance the efficacy of anti‐programmed cell death ligand 1 (PD‐L1) and anti‐PD‐1 drugs through their immunomodulatory role,^34^, ^35^ while PD‐1 inhibitors can reduce tumor angiogenesis; the combination of the two has a synergistic antitumor effect.^36^

In addition, multiple tumors are closely related to patient recurrence and survival. In the above studies, HCC patients with >3 liver tumors could benefit more from TACE with lenvatinib.^15^ As shown in our previous study, tumor size was an important factor for patient survival, differing by the cutoff value of the maximum tumor diameter.^15^ PVTT type III/IV represents a tumor thrombus in the main portal vein, and the superior mesenteric vein or the inferior vena cava, respectively, and this type of classification is generally considered severe and has a poor prognosis.^26^ In our previous study, subgroup analysis showed that TACE combined with lenvatinib was more effective than TACE combined with sorafenib in PVTT I/II, whereas no such difference was observed in PVTT III/IV.^15^ In another study, the results were similar after using propensity score matching.^16^ In addition, a Japanese study found lenvatinib to be superior to sorafenib in HCCs with PVTT Vp3/4 (main portal vein or primary branch tumor thrombus).^37^ And in IMbrave150 study, the analysis of 73 HCCs with PVTT Vp4 demonstrated that atezolizumab combined with bevacizumab was more effective than sorafenib.^19^ These studies have shown that TKI‐based combination regimens are widely used for HCC with various types of PVTT and with good results.

The safety profile of TACE combined with lenvatinib and camrelizumab observed in this study is consistent with the previously reported AEs of TACE, lenvatinib or camrelizumab alone.^14^, ^15^, ^16^, ^17^, ^20^, ^21^, ^23^, ^24^ Although many patients experienced at least one AE, most of the experienced AEs were mild to moderate. RCCEP is a common AE of camrelizumab, as shown in previous studies, but only 10 cases were observed in this study, which may be due to the immunomodulatory effect of lenvatinib on the tumor microenvironment. Although the incidence of Grade 3 hypertension was high, it was controlled with either anti‐hypertensive agents or dose modification. A small proportion of patients developed serious AEs such as hepatic encephalopathy, thrombocytopenia and immune‐related myocarditis, but they improved after the discontinuation of camrelizumab. Given that a continuous lenvatinib treatment will increase the survival benefit of patients, the dose of lenvatinib should be adjusted as much as possible rather than its interruption or substitution with other drugs. In conclusion, the triple combination was well tolerated with manageable toxicity.

This study has several limitations, including the single‐arm design and the small sample size, which preclude more analysis and may bias toward patients who can benefit from the therapy. This study was conducted at three centers in Beijing, China but geographic variation in patients may affect treatment outcomes. Therefore, large‐scale multicenter prospective trials with randomized controls are needed for validation.

## 5 CONCLUSION

TACE combined with lenvatinib and camrelizumab achieved excellent efficacy in HCC patients with all types of PVTT. Importantly, the comprehensive therapy did not add other treatment‐related toxicities, and most adverse reactions could be controlled by dose adjustment and symptomatic treatment.

## AUTHOR CONTRIBUTIONS

**Xiaomi Li:** Data curation (equal); project administration (equal); writing – original draft (equal). **Xiaoyan Ding:** Data curation (equal); project administration (equal); writing – original draft (equal). **Mei Liu:** Data curation (equal); project administration (equal); writing – original draft (equal). **Jingyan Wang:** Formal analysis (equal); writing – review and editing (equal). **Wei Sun:** Formal analysis (equal); writing – review and editing (equal). **Ying Teng:** Formal analysis (equal); writing – review and editing (equal). **Yawen Xu:** Software (equal); supervision (equal). **Hongxiao Wu:** Validation (equal); visualization (equal). **Wendong Li:** Investigation (equal); methodology (equal). **Lin Zhou:** Conceptualization (equal); project administration (equal). **Jinglong Chen:** Conceptualization (equal); project administration (equal).

## CONFLICT OF INTEREST STATEMENT

All authors declare no conflicts of interest.

## ETHICS STATEMENT

The study was conducted according to the guidelines of the Declaration of Helsinki, and approved by the Ethics Committee of Beijing Ditan Hospital, Capital Medical University (BJDT CMU IRB), (ethic code: JDLKZ 2021–003‐02).

## Data Availability

All data generated or analyzed during this study are included in this published article.
